# Unsupervised machine learning analysis of optical coherence tomography radiomics features for predicting treatment outcomes in diabetic macular edema

**DOI:** 10.1038/s41598-025-96988-3

**Published:** 2025-04-18

**Authors:** Xuemei Liang, Shaozhao Luo, Zhigao Liu, Yunsheng Liu, Shinan Luo, Kaiqing Zhang, Li Li

**Affiliations:** 1 Department of Ophthalmology, Aier Eye Hospital, Jinan University, No, 191, Huanshi Middle Road, Yuexiu District, Guangzhou, 510071 Guangdong People’s Republic of China; 2 Department of Ophthalmology, Nanning Aier Eye Hospital, No, 63, Chaoyang Road, Xingning District, Nanning, 530012 Guangxi Zhuang Autonomous Region People’s Republic of China; 3 Department of Ophthalmology, Jinan Aier Eye Hospital, No. 1916, Erhuan East Road, Licheng District, Jinan City, Shandong Province People’s Republic of China; 4 Department of Ophthalmology, Cenxi Aier Eye Hospital, No. 101, Yuwu Avenue, Cenxi City, Wuzhou City, Guangxi Zhuang Autonomous Region People’s Republic of China

**Keywords:** Diabetic macular edema, Anti-vascular endothelial growth factor, OCT-omics feature, Machine learning, Treatment outcomes

## Abstract

This study aimed to identify distinct clusters of diabetic macular edema (DME) patients with differential anti-vascular endothelial growth factor (VEGF) treatment outcomes using an unsupervised machine learning (ML) approach based on radiomic features extracted from pre-treatment optical coherence tomography (OCT) images. Retrospective data from 234 eyes with DME treated with three anti-VEGF therapies between January 2020 and March 2024 were collected from two clinical centers. Radiomic analysis was conducted on pre-treatment OCT images. Following principal component analysis (PCA) for dimensionality reduction, two unsupervised clustering methods (K-means and hierarchical clustering) were applied. Baseline characteristics and treatment outcomes were compared across clusters to assess clustering efficacy. Feature selection employed a three-stage pipeline: exclusion of collinear features (Pearson’s r > 0.8); sequential filtering through ANOVA (*P* < 0.05) and Boruta algorithm (500 iterations); multivariate stepwise regression (entry criteria: univariate *P* < 0.1) to identify outcome-associated predictors. From 1165 extracted radiomic features, four distinct DME clusters were identified. Cluster 4 exhibited a significantly lower incidence of residual/recurrent DME (RDME) (34.29%) compared to Clusters 1–3 (*P* = 0.003, *P* = 0.005 and *P* = 0.002, respectively). This cluster also demonstrated the highest proportion of eyes (71.43%) with best-corrected visual acuity (BCVA) exceeding 20/63 (*P* = 0.003, *P* = 0.005 and *P* = 0.002, respectively). Multivariate analysis identified *logarithm_gldm_DependenceVariance* as an independent risk factor for RDME (*OR* 1.75, 95% CI 1.28–2.40; *P* < 0.001), while *Wavelet-LH_Firstorder_Mean* correlated with worse visual outcomes (*OR* 8.76, 95% CI 1.22–62.84; *P* = 0.031). Unsupervised ML leveraging pre-treatment OCT radiomics successfully stratifies DME eyes into clinically distinct subgroups with divergent therapeutic responses. These quantitative features may serve as non-invasive biomarkers for personalized outcome prediction and retinal pathology assessment.

## Introduction

Diabetic macular edema (DME) is a highly common and severe vision-threatening complication of diabetic retinopathy (DR)^1^. A systematic review and meta-analysis conducted in 2020 projected that the global prevalence of DME among adults is expected to escalate from 18.83 million to 23.50 million by 2030 and to 28.61 million by 2045, reflecting an increase of 24.8% and 51.9%, respectively^2^. DME profoundly impacts overall quality of life and imposes considerable socioeconomic burdens on healthcare systems.

Optical coherence tomography (OCT) has emerged as a mainstay technology for DME diagnosis and monitoring progression and prognostication throughout the course of intravitreal injection therapy^3,4^. The advent of intravitreal anti-vascular endothelial growth factor (anti-VEGF) therapy has revolutionized DME management, resulting in decreasing retinal capillaries permeability and the possibility of reversing vision loss^5,6^. However, challenges such as DME recurrence and poor treatment response remain. A substantial proportion (35%-65%) of DME cases exhibit an inadequate response to anti-VEGF therapy, leading to the diagnosis of persistent DME^7^. Furthermore, the frequent relapses of DME within a short period can culminate in persistent disease. Previous OCT-based studies have explored various parameters, such as hyper-reflective dots and large intraretinal cysts, as potential predictors of treatment response^8,9^. Besides, presence and extent of disorganization of the inner retinal layers (DRIL) and the integrity of ellipsoid zone (EZ) or external limiting membrane (ELM) were considered to a possible prognostic factor of vision^10,11^. However, most studies about predictors were qualitative (presence or absence) or semi-quantitative (measuring only length or proportion), limiting precision and reproducibility. If a non-invasive and quantitative solution could be developed to more effectively predict the outcomes of anti-VEGF treatment for DME, it would be a powerful tool in modern medicine.

Radiomics, a field introduced in 2012, involved the extraction and analysis of high-dimensional and high-throughput quantitative imaging features, aims to enable precision medicine by developing predictive and prognostic models^12–14^. Machine learning (ML) has been widely applied and have an extraordinary capacity to provide an accurate and efficient system for DME screening and diagnosis^15^. However, the combination of ML and radiomics analysis in ophthalmology field is rare until 2024. Chinese scholars Meng et al., introduced the concept of OCT-omics, which integrates OCT imaging with radiomics analysis to comprehensively assess anti-VEGF treatment response in DME patients using supervised learning^16^. Looking ahead, we anticipate that the synergy between OCT-omics and ML is poised to unlock new frontiers in ophthalmic medicine, especially in full-stack analysis of DME, improving patient outcomes while reducing treatment cost.

In contrast, our study employs an unsupervised ML approach to detect and categorize the underlying heterogeneity within DME. Unsupervised ML is a powerful analytical tool that enables researchers to gain insights into the underlying data distribution, thereby capturing disease heterogeneity, identifying novel biomarkers, and elucidating the underlying structures and relationships within the data^17^. Nevertheless, to our best knowledge, there has been no work done to identify radiomic subtypes of DME using an unsupervised method. We hypothesized that using unsupervised ML to analyze radiomic data extracting from pre-treatment OCT of DME patients, could well distinguish patients with different anti-VEGF treatment responses or visual outcomes.

## Methods

### Ethics approval and consent to participate

Ethical approval (No. NNAIER2019IRB01 and JNAIER 2019-02) for this study was obtained from the Ethics Committee of the Nanning Aier Eye Hospital and Jinan Aier Eye Hospital. Written informed consent was waived for the retrospective study. Patient data were anonymized and treated confidentially to ensure privacy and comply with data protection regulations.

### Participant cohort

This observational clinical study enrolled patients diagnosed with DME between January 2020 and March 2024 at Nanning Aier Eye Hospital and Jinan Aier Eye Hospital. Clinical data were reviewed from electronic medical records, and OCT images were acquired from the picture archiving system.

**Fig. 1:**
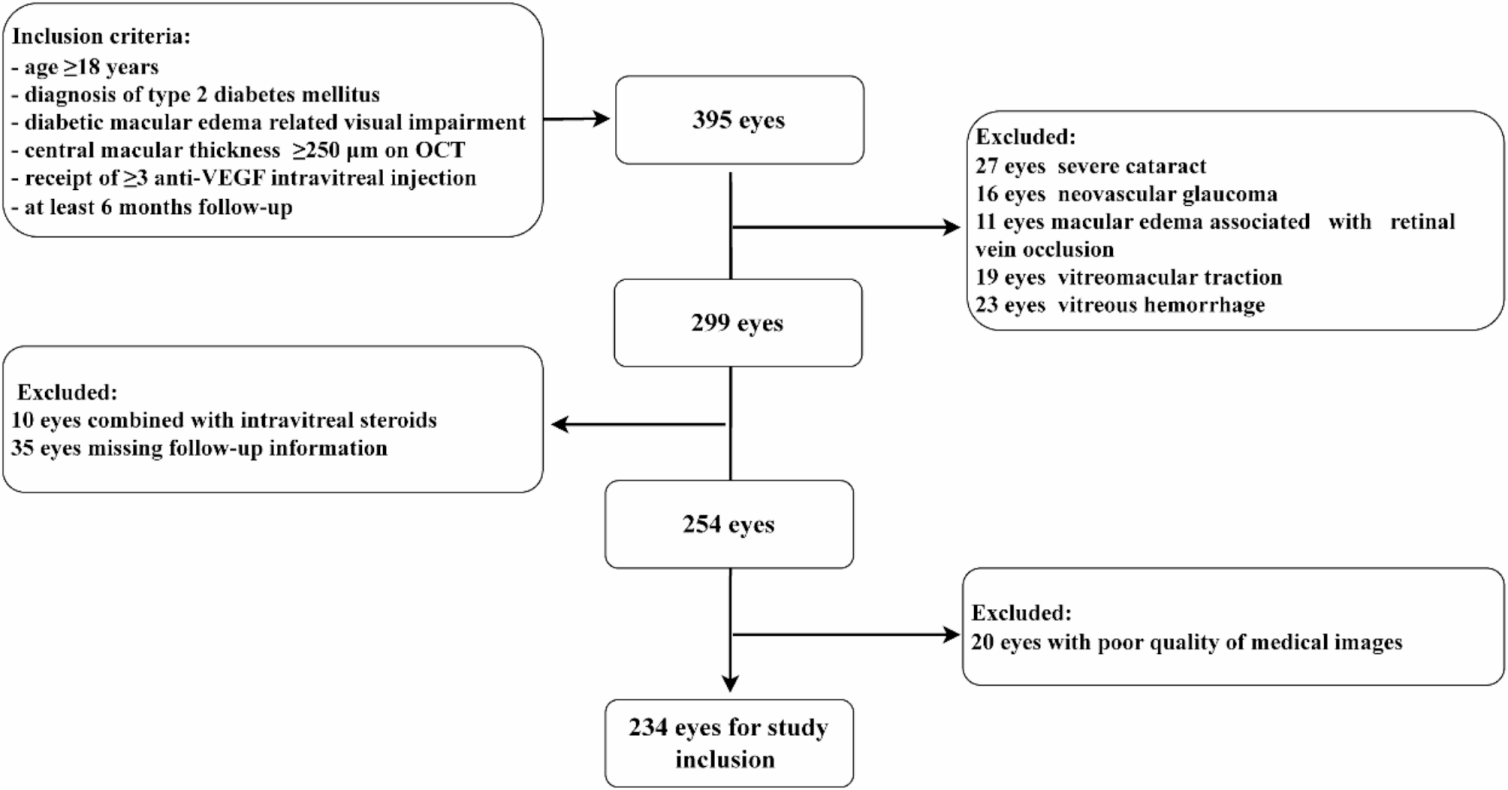
Flowchart of eligibility criteria and data selection.

The inclusion and exclusion criteria are detailed in the workflow chart (Fig. 1). Out of 395 eyes with DME, 234 were deemed eligible for the study, with 161 being excluded. All patients included in this study were treatment-naïve to intravitreal anti-VEGF therapy. Central macular thickness (CMT) was determined as the average thickness from the internal limiting membrane to the retinal pigment epithelium within the central 1-mm diameter area of the Early Treatment Diabetic Retinopathy Study (ETDRS) grid. Anti-VEGF treatments administered included ranibizumab (0.5 mg/0.05 mL, Novartis Pharma AG, Basel, Switzerland), aflibercept (2.0 mg/0.05 mL, Bayer Healthcare Pharmaceuticals, Berlin, Germany), and Conbercept (0.5 mg/0.05 mL, Kanghong Biotech Co., Ltd., Chengdu, China). For patients with bilateral DME, both eyes were included in the study provided that they met all the established inclusion and exclusion criteria.

### Clinical variables

The following clinical variables were collected at baseline: demographic data, including age and gender; detailed medical information such as duration and type of DM, systemic hypertension, hyperlipidemia, and insulin use, stage of DR (non-proliferative DR [NPDR] or proliferative DR [PDR]), history of vitrectomy or cataract surgery, intravitreal injections, presence of pan-retinal photocoagulation (PRP), and type of intravitreal injection drugs; laboratory data including glycated hemoglobin (HbA1c) and serum creatinine; and ophthalmic data including BCVA, intraocular pressure (IOP), and CMT at each visit. The baseline BCVA was converted to the logarithm of the minimum angle of resolution (log MAR) for reporting purposes, following the methodology of a previous study, when necessary^18^.

### OCT image acquisition and region of interest segmentation

OCT images for all subjects in this study were obtained using the Heidelberg Spectralis OCT system (Heidelberg Engineering, Germany). An average number of 80 b-scans were utilized to improve the signal-to-noise ratio. Region of interest (ROI) segmentation and feature extraction were performed on JPG-formatted B-scan OCT images (6 × 6 mm) centered on the fovea. ROI encompassing the inner retinal layers (from the internal limiting membrane to the external limiting membrane), was manually delineated by an experienced ophthalmologist (YS.L., with 10 years of experience) utilizing the LabelMe annotation tool, an open-source tool designed for image labeling and annotation (available at https://github.com/wkentaro/labelme). The initial segmentation results were subsequently validated by a senior retina specialist (L.L., with 30 years of experience) and re-labeled in case of any discrepancy to create final ROI. To quantify consistency, the primary specialist re-analyzed a random subset of 20% of OCT images one month later. The intraclass correlation coefficient (ICC) for radiomic feature extraction was 0.94 (95% CI: 0.89–0.97), demonstrating excellent intra-rater reliability.

### Radiomics feature extraction and selection

A total of 234 radiomic features were initially extracted from OCT-derived ROI using a Python-based computational framework implementing the PyRadiomics toolkit. Feature categories included: 1) 2D shape-based descriptors (n = 9); 2) first-order intensity statistics (n = 18); 3) gray-level co-occurrence matrix (GLCM) features (n = 24); 4) gray-level run length matrix (GLRLM) features (n = 15); 5) gray-level size zone matrix (GLSZM) features (n = 16); 6) gray-level dependence matrix (GLDM) features (n = 14); 7) neighboring gray tone difference matrix (NGTDM) features (n = 5). Post-image transformation processing yielded an additional 1,063 derived features through seven filter families: exponential (n = 93), gradient magnitude (n = 92), local binary pattern (LBP, n = 41), logarithmic (n = 279), wavelet decomposition (n = 372), square root (n = 93), and squaring operators (n = 93). This comprehensive workflow yielded a total of 1,165 quantitative radiomic features. All extracted features conformed to the Image Biomarker Standardization Initiative (IBSI) guidelines^19^, ensuring computational reproducibility.

### Principal

**Fig. 2:**
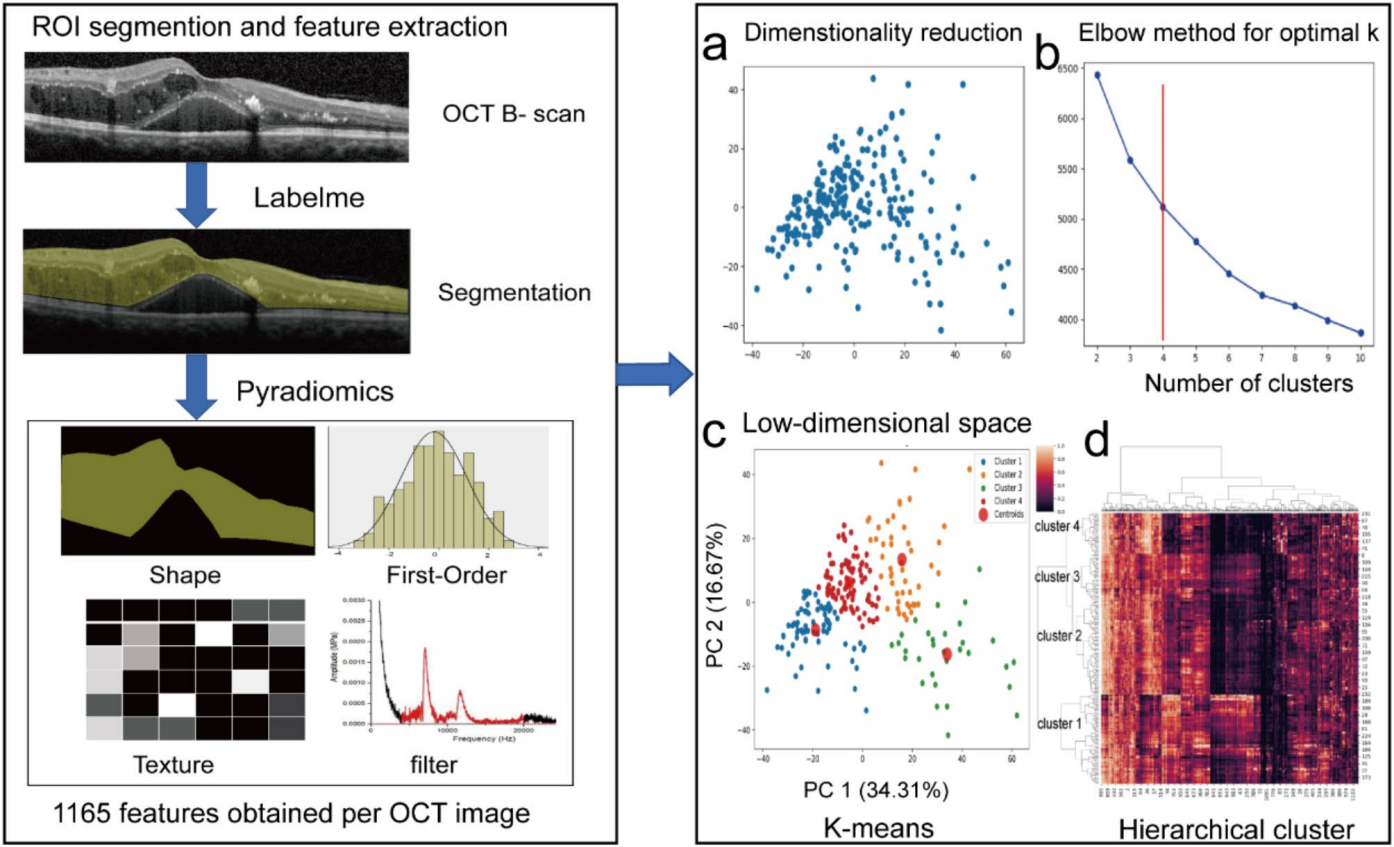
Overview of the proposed analysis. (left panel) ROI segmented from the inner limiting membrane to the external limiting membrane and feature extraction. (right panel) PCA to position subjects according to their similarity through dimensionality reduction, The first two principal component of the PCA explained 50.98% of the variance of the data (**a**). Elbow plot indicating the optimal number of clusters based on the “levelling-off” point (k) (**b**). The scatter plot represents the distribution of final four cluster solution (**c**) and the hierarchical heat-map show four clusters of patients with different expressions of radiomic features based on the dendrogram (**d**). ROI, region of interest; PCA, principal component analysis; OCT, optical coherence tomography.

Principal component analysis (PCA) was implemented for dimensionality reduction and noise suppression in the high-dimensional radiomic dataset. This multivariate technique excels at identifying latent variance sources while effectively mitigating overfitting risks through orthogonal transformation^20^. Prior to PCA, feature matrices were standardized via z-scored using Scikit-learn preprocessing module (v1.1.2) in Python 3.11.6. PCA execution and component selection employed Matlab version 9.5, with optimal principal components (PCs) rigorously determined through Horn’s parallel analysis. This statistical Monte Carlo simulation framework compares observed-data eigenvalues against 1,000 permuted null datasets with identical dimensions. Sixteen PCs satisfying the eigenvalue retention criterion (observed > 95th percentile of random distribution) were selected, capturing 90.03% cumulative explained variance for subsequent analyses. The first two PCs demonstrated dominant variance contributions (50.98% combined), with spatial feature distributions visualized in Fig. 2C.

### Unsupervised clustering analysis

Unsupervised clustering analysis, a data-driven methodology for determining the optimal number of clusters (k) within a data-set, relies on an iterative process that capitalizes on the similarity among radiomics features^21^. In our study, we adopted a hybrid approach, combining hierarchical clustering (Ward method) and the K-means algorithm to determine the optimal number of clusters based on both original and filtered radiomic features. Ultimately, our analysis revealed four distinct clusters of DME. The unsupervised ML process involved a sequential flow, as depicted in Fig. 2.

### Clustering stability validation (Bootstrap method)

**Fig. 3:**
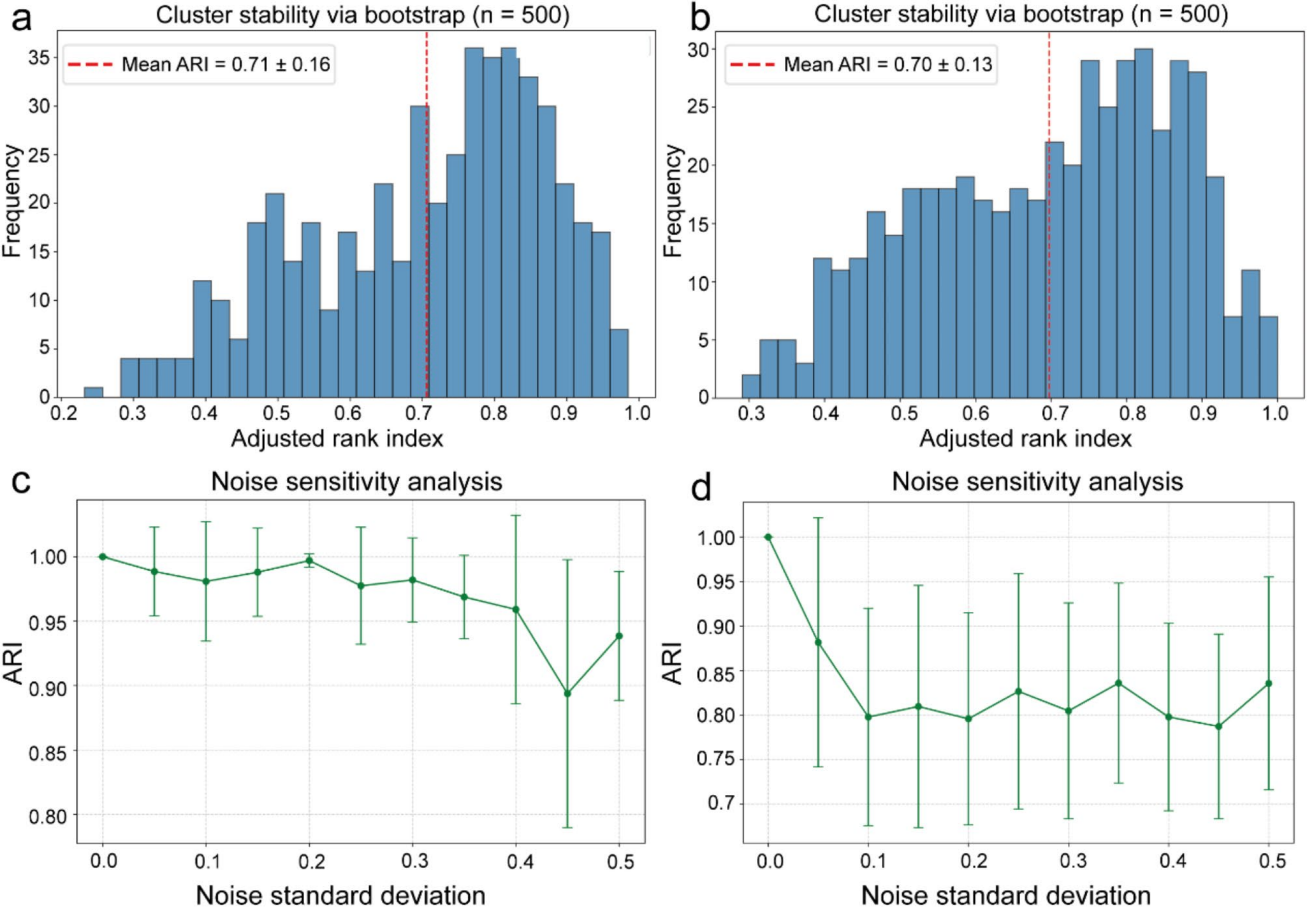
Clustering stability validation. Bootstrap ARI distribution for RDME (**a**) and visual prognosis (**b**): Distribution of cluster consistency across 500 bootstrap iterations. The red dashed line denotes the mean ARI value. Noise sensitivity curve for RDME (**c**) and visual prognosis (**d**): Cluster stability under incremental imaging noise. (**c**) Shows high stability across different noise levels, particularly at low and medium levels, with ARI values remaining above 0.9. (**d**) Indicates slight lower stability, especially at higher noise levels, where ARI values drop below 0.85.

To evaluate the robustness of radiomics feature clustering results, we employed the bootstrap resampling method^22^ (Fig. 3). The specific workflow was as follows: First, resampling: We generated 500 bootstrap datasets by randomly drawing equivalent samples with replacement from the original dataset (n = 234). Second, repeated clustering: For each bootstrap dataset, we replicated the same analytical pipeline—PCA dimensionality reduction (retaining 16 principal components) followed by K-means clustering (k = 4)—to derive new cluster labels. Finally, consistency assessment: The agreement between bootstrap-derived clusters and the original clustering was quantified using the Adjusted Rand Index (ARI). The ARI ranges from [-1, 1], where 1 indicates perfect concordance and 0 represents random-level agreement.

### Study endpoint

The primary endpoint was the presence of residual or short-term recurrent DME (RDME). Residual DME was defined as insufficient CMT reduction following three intravitreal anti-VEGF treatments, adhering to the criteria proposed by Sorour et al.^7^: a reduction in CMT of < 10% for retinal thickness ≤ 400 μm, < 15% for 401–500 μm, < 20% for 501–600 μm, and < 25% for > 600 μm. Short-term recurrent DME was defined as the re-emergence of intraretinal or sub-foveal fluid within 6 months post-treatment in eyes that had achieved complete anatomical and preferably functional resolution after three intravitreal anti-VEGF treatments.

The secondary endpoint was the BCVA at 6 months post-treatment. BCVA was categorized into two groups: low vision (BCVA ≤ 20/63) and normal vision (BCVA > 20/63).

### Statistical analysis

In this study, cluster analysis was utilized to stratify the cohort into 4 distinct subgroups. After that, statistical analyses were performed to compare the baseline characteristics and treatment outcomes among the identified subgroups. For continuous variables, the Kruskal–Wallis H test was employed, and Dunn’s test for multiple comparisons for significant results, while the chi-square test was used for categorical variables. Continuous data were presented as means with standard deviations (SD) or median with interquartile range (IQR), and categorical variables were reported as counts and percentages. To assess the normality of distribution, the Shapiro–Wilk test was applied. Feature selection was performed through a three-stage process. First, highly collinear features (Pearson correlation coefficient > 0.8) were excluded to mitigate redundancy. Subsequently, ANOVA was applied to identify OCT-derived radiomic features with significant intergroup differences. Then significant ANOVA results were subjected to further selection using Boruta algorithm (500 iterative). This process aims to identify variables that significantly contribute to the predictive outcomes. Finally, the retained features underwent univariate analysis followed by multivariate stepwise regression to determine their independent associations with treatment outcomes. The multivariate analysis started with significant variables from the univariate analysis (*P* < 0.1) and used backward step-down selection. All statistical analyses and data visualizations were performed using R (version 4.4.2) and JD_DCPM (V6.03, Jingding Medical Technology Co., Ltd.) and Python software (version 3.11). All hypothesis tests were two sided, and *P* < 0.05 indicated a significant difference.

## Results

### Summary of study design and participant characteristics

The study process encompassed the formulation of clinical concerns, screening of clinical data, acquisition of medical images, segmentation of ROI^23^, unsupervised cluster analysis, cluster comparison, radiomics features selection and correlation analysis^20^.

### Unsupervised ML on OCT-omics features

**Fig. 4:**
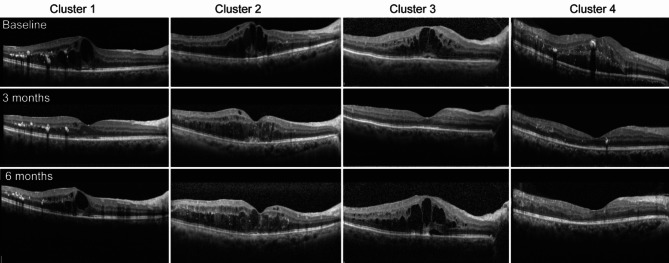
Radiomic clustering analysis of OCT Images in DME prognosis. All clusters share five key radiomic features: Risk factors for RDME: *logarithm_gldm_DependenceVariance (LogGDV)*. Risk factors for visual prognosis: *log-sigma-5-mm-3D_glszm_LargeAreaHighGrayLevelEmphasis (LogSAHG), wavelet-LH/HH_firstorder_Mean/Median (LH-Mean/HH-Median).* Protective factor for visual prognosis: *log-sigma-5-mm-3D_glrlm_LongRunHighGrayLevelEmphasis (LogRHE).*

In our analysis of all computed radiomics features, consensus clustering demonstrated a significant reduction in the relative change of the area under the conditional density function curve, specifically when the number of clusters (k) was set to 4. Baseline clinical characteristics and treatment outcomes were compared between the clusters of patients (Table 1). Cluster 1 contained 122 eyes from 99 patients, Cluster 2 contained 45 eyes from 39 patients, Cluster 3 contained 32 eyes from 28 patients, and Cluster 4 contained 35 eyes from 28 patients. Example images were selected from eyes near the center of each cluster and included for illustrative purposes (Fig. 4).

**Table 1:** Baseline clinical characteristics and outcomes variables of the four clusters identified by clustering analysis on OCT-omics features before anti-VEGF.

Characteristics	Overall	Cluster 1	Cluster 2	Cluster 3	Cluster 4	*P* value
Participant level	N = 131	N = 98	N = 39	N = 28	N = 28	
Age (y), Mean ± SD	59.27 ± 9.91	58.19 ± 9.71	61.09 ± 8.92	60.88 ± 11.07	59.23 ± 10.61	0.284
Sex, male/female	71 / 60	59 / 39	21 / 18	13 / 15	14 / 14	0.353
DM course (y), M (Q₁, Q₃)	10.00 (5.00, 12.75)	10.00 (5.00,10.00)	10.00 (5.00,16.00)	9.39 (5.00,20.00)	10.00 (7.50,10.00)	0.824
HbA1C (%), M (Q₁, Q₃)	7.29 (6.66, 8.65)	7.05 (6.49,7.52)	7.52 (7.05,7.52)	7.23 (6.26,7.52)	7.05 (6.42,7.51)	0.345
Insulin usage, n (%)	51 (38.93)	58 (47.54)	21 (46.67)	10 (31.25)	16 (45.71)	0.420
Hypertension, n (%)	58 (49.15)	63 (51.64)	18 (40.00)	16 (50.00)	18 (51.43)	0.595
Hyperlipidemia, n (%)	49 (37.40)	27 (22.13)	9 (20.00)	6 (18.75)	7 (20.00)	0.971
Creatinine, (mmol/l) M (Q₁, Q₃)	84.80 (66.50, 108.25)	84.10 (63.60,109.00)	81.90 (63.00,106.00)	78.25 (64.95,109.00)	88.00 (76.45,108.00)	0.669
Eye level	N = 234	N = 122	N = 45	N = 32	N = 35	
Laterality (right/left)	117 / 117	58 / 64	27 / 18	14 / 18	18 / 17	
DR stages (NPDR/PDR)	178 / 56 (76.07)	91/31 (74.59)	29/16 (64.44)	29/3 (90.62)	29/6 (82.86)	**0.044**
Previous PRP	41 (17.52)	19 (15.57)	12 (26.67)	6 (18.75)	4 (11.43)	0.242
Previous surgery	59 (25.21)	33 (27.05)	14 (31.11)	5 (15.62)	7 (20.00)	0.375
BCVA (Log MAR), M (Q₁, Q₃)	0.50 (0.30, 0.70)	0.50 (0.30,0.70)	0.50 (0.30,0.80)	0.50 (0.20,0.70)	0.40 (0.30,0.55)	0.533
IOP (mmHg), M (Q₁, Q₃)	14.00 (12.43, 17.00)	15.00 (13.00,17.95)	14.00 (13.00,17.00)	14.75 (11.93,17.12)	13.00 (12.00,16.50)	0.554
CMT (μm), M (Q₁, Q₃)	408.50 (299.25, 588.50)	386.00 (301.00,528.25)	510.00 (347.00,679.00)	542.50 (397.75,640.00)	286.00 (262.50,455.00)	** < 0.001**
Medications, n (%)						0.171
Ranibizumab	95 (40.60)	51 (41.80)	15 (33.33)	9 (28.12)	20 (57.14)	
Conbercept	63 (26.92)	35 (28.69)	14 (31.11)	8 (25.00)	6 (17.14)	
Aflibercept	76 (32.48)	36 (29.51)	16 (35.56)	15 (46.88)	9 (25.71)	
Outcome variables						
RDME, n (%)						**0.017**
Yes	134 (57.26)	72 (59.02)	31 (68.89)	19 (59.38)	12 (34.29)	
No	100 (42.74)	50 (40.98)	14 (31.11)	13 (40.62)	23 (65.71)	
Visual prognosis, n (%)						**0.007**
≤ 20/63	128 (54.70)	70 (57.38)	27 (60.00)	21 (65.62)	10 (28.57)	
> 20/63	106 (45.30)	52 (42.62)	18 (40.00)	11 (34.38)	25 (71.43)	

DM, diabetes mellitus; DR, diabetic retinopathy; HbA1c, glycated haemoglobin; BCVA, best corrected visual acuity; CMT, central macular thickness; IOP, intraocular pressure; DME, diabetic macular edema; RDME, residual / recurrent DME.

*Fisher exact test or chi-square test were used for categorical variables (sex, insulin usage, hypertension, hyperlipidemia, DR stages, previous PRP, previous surgery, medications, residual / recurrent DME, visual prognosis); Kruskal–Wallis test was used for continuous variables (age, DM course, HbA1C, creatinine, Log MAR BCVA, IOP CMT).

Significant values are in [bold].

Cluster 1 (Recurrent DME, Moderate Prognosis): *LogGDV* = 5.75, *LogRHE* = 4,259.81, *LogSAHG* = 43.4 M, *LH-Mean* = -0.38, *HH-Median* = 0.12. Case: 56 M, right eye (RE), baseline CMT 630 μm, BCVA 20/50. Post 3 anti-VEGF injections (4 months), recurrence with CMT↑498 μm and BCVA↓20/63. Cluster 2 (Residual DME, Severe Prognosis): *LogGDV* = 8.07, *LogRHE* = 2,996.11, *LogSAHG* = 28.8 M, *LH-Mean* = -0.41, *HH-Median* = 0.14. Case: 57F (RE), baseline CMT 638 μm, BCVA 20/100. Persistent DME post-treatment (CMT 468 μm, BCVA 20/80). Cluster 3 (Recurrent DME, Severe Prognosis): *LogGDV* = 8.53, *LogRHE* = 2,731.37, *LogSAHG* = 45.3 M, *LH-Mean* = -0.39, *HH-Median* = 0.21. Case: 60 M (RE), baseline CMT 592 μm, BCVA 20/125. Recurrence at 6 months post-treatment (CMT↑719 μm, BCVA↓20/200). Cluster 4 (Milder or treatment-responsive DME): *LogGDV* = 3.90, *LogRHE* = 2,934.35, *LogSAHG* = 26.6 M, *LH-Mean* = -0.59, *HH-Median* = 0.09. Case: 41 M (RE), baseline CMT 598 μm, BCVA 20/80. Full recovery post-treatment (CMT↓198 μm, BCVA↑20/40).

### Baseline clinical characteristics compared by clusters

A total of 234 eyes from 131 patients were included, and 103 (78.63%) patients had bilateral eyes involved. Demographic data, ocular characteristics, and treatment outcomes, stratified by cluster, are detailed in Table 1. The mean age of the patient cohort was 59.05 ± 10.39 years, with 71 (54.20%) being male and 60 (45.80%) female. The median duration of DM was 10.0 years (IQR, 5.0 to 12.75 years). Among the 234 eyes, 76.07% were classified as NPDR. The mean initial CMT was 408.0 μm (IQR: 299.25 to 588.50 μm). Intergroup comparisons of baseline characteristics across clusters indicated that age, sex, DM duration, HbA1c, insulin use, hypertension, hyperlipidemia, and serum creatinine levels were approximately the same trend across the four clusters.

Eye-level variables were compared across different clusters. Chi-squared tests revealed statistically significant differences in DR stage. Cluster 3 had the highest proportion of patients with NPDR at 90.62%, which was significantly higher than in Clusters 1 and 2 (*P* = 0.042 and *P* = 0.009, respectively). Besides, Cluster 4 consisted of the lowest CMT values, which were significantly lower than those in Cluster 2 and 3 (both *P* < 0.001). However, no significant differences were observed in prior PRP, intraocular surgery, or the distribution of the initial three medication types among the four clusters. Additionally, no significant differences were observed in either initial Log MAR BCVA or IOP.

### Treatment outcomes compared by clusters

Among the 234 eyes analyzed, 134 (57.26%) exhibited RDME, while 128 (54.7%) demonstrated suboptimal visual acuity (BCVA ≤ 20/63), with significant outcome heterogeneity across clusters (all *P* < 0.05). Cluster stratification revealed distinct clinical profiles. Cluster 1 (N = 122, 51.14%) exhibited intermediate characteristics with 59.02% RDME and 57.38% BCVA ≤ 20/63, defining a moderate severity subgroup. In contrast, Cluster 2 (N = 45, 19.23%) demonstrated the highest RDME prevalence (68.89%, *P* = 0.002 vs Cluster 4), whereas Cluster 3 (N = 64, 13.67%) had the poorest visual outcomes (65.62% with BCVA ≤ 20/63, *P* = 0.002 vs Cluster 4). Collectively representing treatment-resistant subgroups (32.91% of cohort). Most notably, Cluster 4 (N = 35, 14.96%) showed the most favorable outcomes, with the lowest RDME incidence (34.29% vs 59.02–68.89% in other clusters; *P* = 0.01, *P* = 0.002 and *P* = 0.04, respectively) and highest BCVA > 20/63 attainment (71.43% vs 34.38–42.62%; *P* = 0.003, *P* = 0.005 and *P* = 0.002, respectively), suggesting treatment-responsive phenotypes.

### Clustering effect evaluation

Bootstrap validation demonstrated moderate-to-high stability in patient subtype classification for RDME (mean ARI = 0.71 ± 0.16, 95% CI: 0.36–0.94) and visual prognosis (mean ARI = 0.70 ± 0.13, 95% CI: 0.37–0.96) (Fig. 3a,b). Further noise sensitivity testing revealed that the ARI remained above 0.9 in Fig. 3c and above 0.8 in Fig. 3d when Gaussian noise with a standard deviation ≤ 0.3 was added, confirming the clustering robustness to imaging noise.

### Significant feature selection workflow

**Fig. 5:**
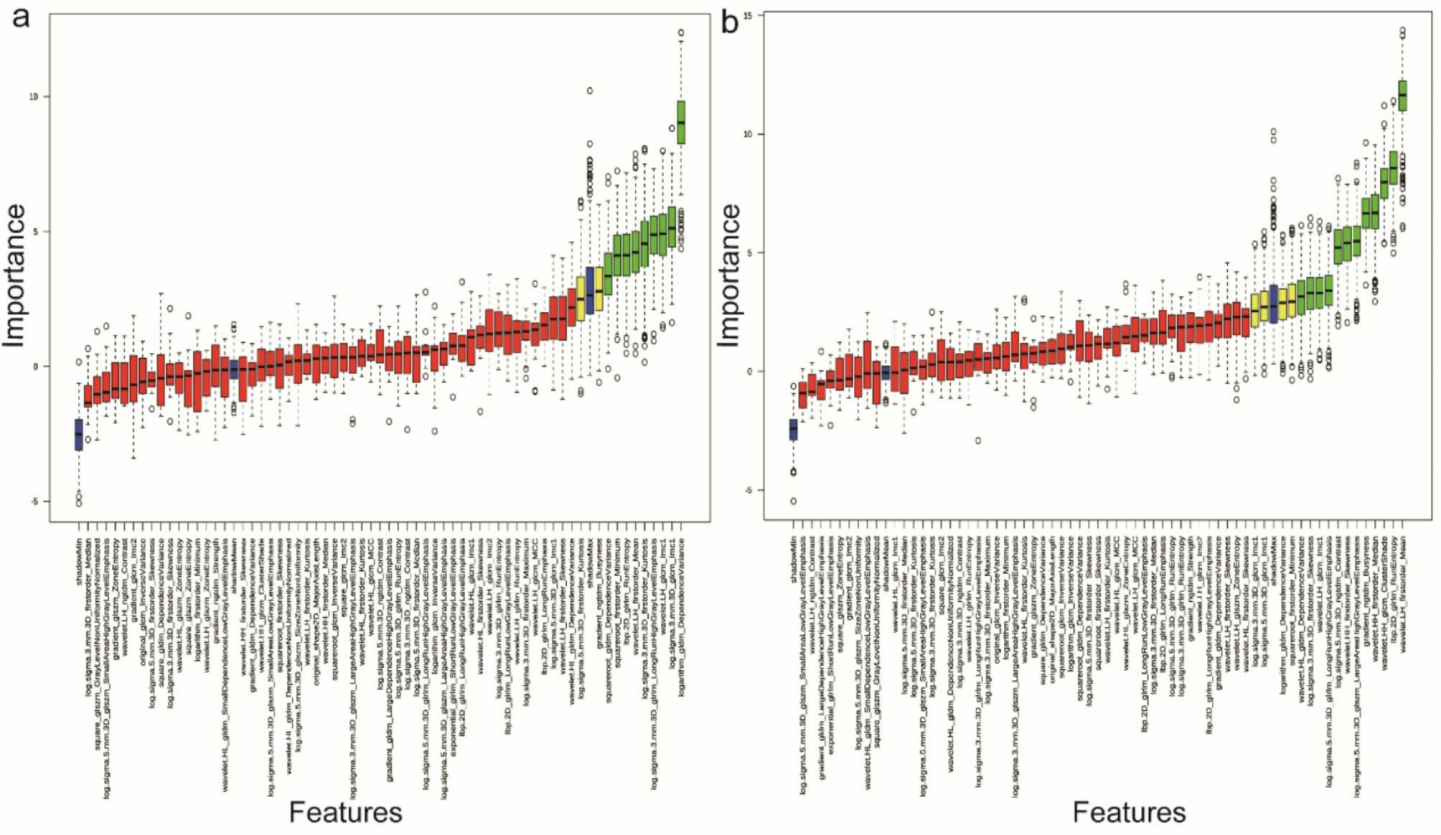
Boruta-driven feature importance ranking for (**a**) residual/recurrent DME (RDME) and (**b**) visual prognosis outcomes. The horizontal axis displays features ranked by ascending importance scores, with vertical axis values quantifying relative feature contribution. Color-coded boxplots denote feature classification: green (confirmed predictors), yellow (tentative candidates), red (irrelevant features), and blue (synthetic shadow features generated for permutation testing).

Three-stage feature selection was performed: (1) Collinearity reduction via Pearson correlation thresholding (|r|> 0.8 excluded), retaining 76 features; (2) ANOVA-based intercluster screening (*P* < 0.05), identifying 64 differentially expressed features; (3) Boruta algorithm (500 iterations) for outcome-specific biomarker identification. The RDME-associated signature comprised nine radiomic biomarkers: *lbp-2D_glrlm_RunEntropy*, *log-sigma3_3D-firstorder_Kurtosis*, *log-sigma3_3D-glcm_Imc1*, *log-sigma3_3D-glrlm_LongRunHighGrayLevelEmphasis*, *logarithm_gldm_DependenceVariance*, *squareroot_firstorder_Minimum*, *squareroot_gldm_DependenceVariance*, *wavelet-LH_firstorder_Mean*, and *wavelet-LH_glcm_Imc1* (Fig. 5a). Similarly, visual prognosis analysis identified twelve biomarkers including *gradient_ngtdm_Busyness*, *lbp-2D_glrlm_RunEntropy*, *log-sigma5_3D-glrlm_LargeAreaHighGrayLevelEmphasis*, *wavelet-HH_firstorder_Skewness*, and *wavelet-HH_glcm_ClusterShade* (Fig. 5b).

### Regression analysis of outcome-associated features

Post-Boruta feature selection, multivariable logistic regression analyses identified OCT-derived biomarkers associated with RDME progression and visual prognosis. Multivariable analysis identified *logarithm_gldm_DependenceVariance* as an independent RDME predictor (*OR* 1.75, 95% CI 1.28–2.40; *P* < 0.001), demonstrating effect size stability versus univariate estimates (Table 2). Visual outcome revealed four significant biomarkers: *log-sigma5_3D-glrlm_LongRunHighGrayLevelEmphasis* showed protective effects against visual decline (*OR* 0.99, 95% CI 0.98–0.99; *P* < 0.001), whereas *log-sigma5_3D-glszm_LargeAreaHighGrayLevelEmphasis* (*OR* 1.01, 95% CI 1.01–1.02; *P* < 0.001) and *wavelet-LH_firstorder_Mean* (*OR* 8.76, 95% CI 1.22–62.84; *P* = 0.031) emerged as risk amplifiers. But *wavelet-LH_firstorder_Median* (*OR* 489.60, 95% CI 22.98–10,433.11; *P* < 0.001) with exceptional effect size (Table 3).

**Table 2:** Univariate and multivariate backward regression for RDME.

Radiomic features	Univariate regression analysis			Multiple regression analysis		
	β	*P*	*OR* (95% *CI*)	*β*	*P*	*OR* (95% *CI*)
*Log-Sigma-3-Mm-3d Glcm Imc1*	22.35	**0.010**	5,100,944,687.76 (208.40 ~ 124,853,968,381,009,888.00)			
*Logarithm Gldm Dependencevariance*	0.66	** < .001**	1.93 (1.44 ~ 2.60)	0.56	** < .001**	1.75 (1.28 ~ 2.40)
*Wavelet-Lh Firstorder Mean*	2.50	**0.001**	12.22 (2.66 ~ 56.22)			
*Wavelet-Lh Glcm Imc1*	19.96	**0.020**	463,857,610.18 (22.89 ~ 9,400,332,532,432,246.00)			
*Log-Sigma-3-Mm-3d Firstorder Kurtosis*	0.05	0.587	1.05 (0.87 ~ 1.27)			
*Log-Sigma-3-Mm-3dGlrlm Longrunhighgraylevelemphasis*	− 0.01	**0.019**	0.99 (0.99 ~ 0.99)			
*Squareroot Firstorder Minimum*	− 0.03	**0.029**	0.97 (0.95 ~ 0.99)			
*Squareroot Gldm Dependencevariance*	0.50	**0.008**	1.66 (1.14 ~ 2.40)			
*Lbp-2d Glrlm Runentropy*	1.09	** < .001**	2.96 (1.55 ~ 5.65)	0.63	0.079	1.88 (0.93 ~ 3.78)

OR, odds ratio; CI, confidence Interval; RDME, residual/recurrent diabetic macular edema.

Significant values are in [bold].

**Table 3:** Univariate and multivariate backward regression for visual prognosis.

Radiomic features	Univariate correlation analysis			Multiple regression analysis		
	*β*	*P*	*OR* (95%CI)	*β*	*P*	*OR* (95% *CI*)
*Gradient Ngtdm Busyness*	0.01	** < .001**	1.01 (1.01 ~ 1.01)			
*Lbp-2d Glrlm Runentropy*	1.83	** < .001**	6.25 (3.06 ~ 12.76)			
*Log-Sigma-3-Mm-3d Firstorder Kurtosis*	− 0.05	0.582	0.95 (0.79 ~ 1.14)			
*Log-Sigma-5-Mm-3d Glrlm Longrunhighgraylevelemphasis*	− 0.01	**0.002**	0.99 (0.99 ~ 0.99)	− 0.01	** < .001**	0.99 (0.99 ~ 0.99)
*Log-Sigma-5-Mm-3d Glszm Largeareahighgraylevelemphasis*	0.01	**0.003**	1.01 (1.01 ~ 1.01)	0.01	** < .001**	1.01 (1.01 ~ 1.01)
*Log-Sigma-5-Mm-3d Ngtdm Contrast*	− 330.77	** < .001**	0.00 (0.00 ~ 0.00)			
*Wavelet-Lh Firstorder Mean*	3.63	** < .001**	37.85 (7.48 ~ 191.67)	2.17	**0.031**	8.76 (1.22 ~ 62.84)
*Wavelet-Lh Glcm Imc1*	22.94	**0.008**	9,185,739,554.05 (400.40 ~ 210,733,824,599,206,784.00)			
*Wavelet-Hh Firstorder Median*	5.54	** < .001**	254.89 (18.18 ~ 3572.86)	6.19	** < .001**	489.60 (22.98 ~ 10,433.11)
*Wavelet-Hh Firstorder Skewness*	− 2.68	** < .001**	0.07 (0.02 ~ 0.28)			
*Wavelet-Hh Glcm Clustershade*	− 6.18	**0.007**	0.00 (0.00 ~ 0.19)			
*Wavelet-Hl Gldm Dependencevariance*	0.70	0.059	2.01 (0.97 ~ 4.16)			

OR: Odds Ratio, CI: Confidence Interval.

Significant values are in [bold].

Table 4 presents five predictive radiomic features for anti-VEGF therapeutic outcomes in DME, with significant intercluster differences (ANOVA, all *P* < 0.05). The feature *logarithm_gldm_DependenceVariance* was specifically associated with RDME risk, while the remaining four features were linked to visual prognosis.

**Table 4:** Five significant radiomic features demonstrated significant differences across the clusters.

Features	Total (n = 234)	Cluster 1 (n = 122)	Cluster 2 (n = 45)	Cluster 3 (n = 32)	Cluster 4 (n = 35)	*P*
*Logarithm Gldm Dependencevariance,* Mean ± SD	6.50 ± 1.00	6.49 ± 0.76	6.68 ± 1.05	7.46 ± 0.85	5.45 ± 0.80	** < .001**
*Log-Sigma-5-Mm-3d Glrlm Longrunhighgraylevelemphasis,* Mean ± SD	3537.19 ± 1324.64	3401.62 ± 1209.24	3914.22 ± 1390.74	2804.43 ± 835.44	4194.92 ± 1573.70	** < .001**
*Log-Sigma-5-Mm-3d Glszm Largeareahighgraylevelemphasis,* Mean ± SD	49,122,063.39 ± 22,073,476.52	47,162,299.23 ± 18,313,664.49	57,991,857.57 ± 33,370,085.48	44,144,352.50 ± 15,341,745.80	49,100,270.20 ± 18,668,434.15	**0.019**
*Wavelet-Lh Firstorder Mean,* Mean ± SD	− 0.44 ± 0.18	− 0.47 ± 0.17	− 0.39 ± 0.17	− 0.28 ± 0.11	− 0.55 ± 0.17	** < .001**
*Wavelet-Hh Firstorder Median,* Mean ± SD	0.12 ± 0.12	0.14 ± 0.09	0.10 ± 0.12	0.14 ± 0.20	0.08 ± 0.04	**0.013**

Significant values are in [bold].

## Discussion

In this exploratory work, unsupervised ML identified four clinically distinct DME subtypes through quantitative analysis of 1,165 pretreatment OCT-derived radiomic features. These subtypes demonstrated significant heterogeneity in anti-VEGF therapeutic response (RDME incidence: 34.29–68.89%) and visual outcomes (BCVA ≤ 20/63: 28.57%–65.62%). Pretreatment OCT radiomics may enable clinically actionable risk stratification, with Cluster 4 (low-RDME/good prognosis) showing 71.43% BCVA > 20/63 versus 34.38%–42.62% in other subtypes. Multivariable regression confirmed *logarithm_gldm_DependenceVariance* as an independent RDME predictor (*OR* 1.75, *P* < 0.001) and *wavelet-LH_firstorder_Mean* as a visual prognosis marker (*OR* 8.76, *P* = 0.031), establishing their potential as OCT-based biomarkers. To our knowledge, this constitutes the first implementation of unsupervised learning for imaging-defined RDME subtyping in DME management.

Unsupervised learning enables data-driven discovery of imaging biomarkers without predefined hypotheses^24^, as evidenced by its successful application in AMD pathophysiology studies^25^. Our radiomic pipeline categorized 234 DME eyes into four prognostic groups along a severity gradient: with Cluster 4 (14.95% of cohort) exhibiting the most favorable profile: 34.29% RDME incidence (vs 59.02%–68.89% in Clusters 1–3, *P* < 0.05) and 71.43% BCVA > 20/63 (vs 34.38%–42.62%, *P* < 0.01), suggesting differential fluid clearance capacity. In contrast, Clusters 2–3 (32.91% combined) represented treatment-refractory phenotypes. These imaging-defined phenotypes—ranging from treatment-responsive (Cluster 4) to refractory (Clusters 2–3)—may reflect underlying pathoanatomical differences in retinal fluid dynamics measurable through CMT variations.

The high-dimensional nature of radiomic data in machine learning pipelines demands rigorous feature selection to prevent model overfitting while preserving biological interpretability. We implemented a three-stage feature selection approach: (1) Pearson correlation-based collinearity elimination (|r|> 0.8); (2) ANOVA filtering (FDR-adjusted *P* < 0.05); (3) Boruta wrapper selection (500 iterations), identifying nine RDME-associated radiomic biomarkers. Multivariable logistic regression confirmed *logarithm_gldm_DependenceVariance*—a GLDM-derived texture metric quantifying local gray-level dependency variance—as the sole independent RDME predictor (*OR* 1.75, 95% CI 1.28–2.40; *P* < 0.001) after controlling for baseline central CMT and DR stage. While the pathophysiological basis of radiomic biomarkers in DME prognosis remains to be fully elucidated, *logarithm_gldm_DependenceVariance* likely captures inner retinal layer textural heterogeneity—a known correlate of structural damage in chronic edema. GLDM-based texture analysis has demonstrated clinical validity across pathologies, with dependence variance metrics correlating with tumor heterogeneity and inflammatory activity in prior oncology and ophthalmology studies^26,27^. In DME, elevated *logarithm_gldm_DependenceVariance* may reflect pathological fluid dynamics—including cystoid spaces and intraretinal hyperreflective foci—that disrupt normal OCT reflectivity patterns^28,29^. These results extend recent OCT radiomics evidence demonstrating that macular texture heterogeneity predicts anti-VEGF nonresponse^16^, with Meng et al.'s supervised model achieving 76.5%-82.4% accuracy in refractory prediction using radiomics features.

Our study advances the visual prognostic utility of OCT radiomics in DME by identifying quantitative biomarkers that synergize with conventional OCT metrics. *log-sigma5_3D-glrlm_LongRunHighGrayLevelEmphasis* demonstrated protective effects against visual decline (*OR* 0.99, 95% CI 0.98–0.99; *P* < 0.001), potentially reflecting inhibition of pathological hyperreflective foci formation. Moreover, *log-sigma5_3D-glszm_LargeAreaHighGrayLevelEmphasis* (*OR* 1.01, 95% CI 1.01–1.02) as risk amplifiers, though its minimal effect size (*OR*≈1) limits immediate clinical translation. *wavelet-LH_firstorder_Median* (OR 489.60, 95% CI 22.98–10,433.11) was independent risk factors associated with worse visual outcomes, with extreme large OR values (e.g., *OR* > 10) suggests strong predictive power, but the confidence interval is extremely wide and may be limited by extreme values or sample size requiring external validation. *wavelet-LH_firstorder_Mean* (*OR* 8.76, 95% CI 1.22–62.84; *P* = 0.031) demonstrated a persistent association with adverse visual outcomes in multivariable logistic regression analyses, though with significant effect size attenuation compared to univariate estimates (β reduction = 40.2%), indicating implied partial confounding by CMT or edema morphological subtypes^30^. Collectively, these OCT-omics features form a multidimensional biomarker panel that captures subclinical heterogeneity through quantitative textural analysis and wavelet-transformed feature extraction with multi-scale decomposition, transcending conventional fluid categorization (diffuse vs. cystoid)^31^ while enabling integration with pathoanatomical subtyping based on biomarker-defined phenotypes. This is exemplified by Cluster 4 (low-RDME/good prognosis) correlating with cystoid-predominant anatomy responsive to anti-VEGF therapy, versus Cluster 3 (65.62% BCVA ≤ 20/63) representing severe prognosis phenotypes with ellipsoid zone disruption. The *log-sigma5_3D* and wavelet-transformed features quantify microstructural changes—including fibrotic remodeling and edema chronification—beyond the resolution of conventional OCT biomarkers. This radiomics-enhanced stratification framework provides actionable insights for personalized DME management, particularly in identifying anti-VEGF refractory cases.

Our radiomic framework augments conventional OCT metrics by decoding subvisual microstructural heterogeneity—textural signatures imperceptible to standard fluid volume analyses. For instance, *wavelet-LH_firstorder_Mean* quantifies localized edema dynamics through high-frequency spatial patterns, while *log-sigma5_3D-glszm_LargeAreaHighGrayLevelEmphasis* maps fibrotic progression via large-area gray-level coherence, aligning with emerging evidence advocating multimodal approaches to optimize DME prognosis and management^32^. This integrative paradigm not only refines phenotypic stratification but also bridges the gap between qualitative clinical observations and quantitative imaging analytics, offering a scalable tool for personalized therapeutic strategies.

This study pioneers in establishing OCT radiomics signatures as predictors of DME therapeutic outcomes, yet several limitations warrant consideration. First, the modest cohort size (n = 234) may limit the generalizability of cluster stability, though our dual clustering approach (K-means with hierarchical validation) mitigated overfitting risks through consensus partitioning. Multicenter prospective validation across diverse ethnic populations and OCT devices (e.g., Spectralis vs ZEISS) is imperative to assess clinical translatability. Second, the retrospective design inherently carries selection bias and unmeasured confounders, particularly regarding treatment interval and follow-up adherence. Nevertheless, this first demonstration of OCT radiomics-guided clustering establishes a paradigm for data-driven DME subtyping in resource-limited settings. Third, the absence of external validation cohorts precludes assessment of model generalizability across diverse populations and OCT platforms, necessitating multicenter replication. Finally, while mathematically defined, radiomic features like *logarithm_gldm_DependenceVariance* lack direct histopathological correlates. Integration with aqueous humor proteomics and single-cell RNA sequencing could elucidate their biological basis in edema resolution pathways.

## Conclusions

Our study establishes that pretreatment OCT radiomics-guided unsupervised learning delineates four clinically actionable DME subtypes with divergent anti-VEGF responses. The *logarithm_gldm_DependenceVariance* (*OR* 1.75) and *wavelet-LH_firstorder_Mean* (*OR* 8.76) emerged as robust biomarkers, enabling early identification of high-risk phenotypes (Clusters 2–3: 32.91% cohort) versus treatment-responsive subtypes (Cluster 4: 14.95%). This paradigm shift from conventional fluid-based classification to radiomic-driven subtyping permits. Future multicenter validations integrating proteomic profiling and angiography OCT metrics are warranted to refine these computational phenotypes into bedside decision tools. By bridging pixel-level heterogeneity with pathobiology, our framework pioneers a precision medicine roadmap for DME management.

## Data Availability

The datasets used in the analyses described in this study are available from the corresponding author on reasonable request.

## Abbreviations

DME: Diabetic macular edema
RDME: Residual or recurrent diabetic macular edema
DR: Diabetic retinopathy
VEGF: Vascular endothelial growth factor
ML: Machine-learning
OCT: Optical coherence tomography
PCA: Principal component analysis
ANOVA: Analysis of variance

## References

[1] Lundeen, E. A. et al. Trends in the prevalence and treatment of diabetic macular edema and vision-threatening diabetic retinopathy among commercially insured adults aged <65 years. *Diabetes Care* **46**, 687–696 (2023). 36637915 10.2337/dc22-1834 PMC10928529

[2] Teo, Z. L. et al. Global prevalence of diabetic retinopathy and projection of burden through 2045: Systematic review and meta-analysis. *Ophthalmology* **128**, 1580–1591 (2021). 33940045 10.1016/j.ophtha.2021.04.027

[3] Virgili, G., Menchini, F. & Casazza, G., et al. Optical coherence tomography (OCT) for detection of macular oedema in patients with diabetic retinopathy. *Cochrane Database Syst. Rev.* **1**, CD008081 (2015). 10.1002/14651858.CD008081.pub3 PMC4438571 25564068

[4] Arrigo, A., Aragona, E., Battaglia Parodi, M. & Bandello, F. Quantitative approaches in multimodal fundus imaging: State of the art and future perspectives. *Prog. Retin Eye Res.* **92**, 101111 (2023). 35933313 10.1016/j.preteyeres.2022.101111

[5] Virgili, G., Parravano, M., Evans, J. R., Gordon, I. & Lucenteforte, E. Anti-vascular endothelial growth factor for diabetic macular oedema: a network meta-analysis. *Cochrane Database Syst. Rev.* **10**, CD007419 (2018). 10.1002/14651858.CD007419.pub6 PMC6517135 30325017

[6] Brown, D. M. et al. Intravitreal aflibercept for diabetic macular edema: 100-week results from the VISTA and VIVID studies. *Ophthalmology* **122**, 2044–2052 (2015). 26198808 10.1016/j.ophtha.2015.06.017

[7] Sorour, O. A. et al. Persistent diabetic macular edema: Definition, incidence, biomarkers, and treatment methods. *Surv. Ophthalmol.* **68**, 147–174 (2023). 36436614 10.1016/j.survophthal.2022.11.008

[8] Cao, J. et al. Prediction of response to anti-vascular endothelial growth factor treatment in diabetic macular oedema using an optical coherence tomography-based machine learning method. *Acta Ophthalmol.* **99**, e19–e27 (2021). 32573116 10.1111/aos.14514

[9] Choi, J., Kim, S. J., Kang, S. W., Hwang, S. & Son, K. Y. Biomarkers determining treatment interval of diabetic macular edema after initial resolution by anti-vascular endothelial growth factor. *Graefes Arch. Clin. Exp. Ophthalmol.* **262**, 421–429 (2024). 37843565 10.1007/s00417-023-06269-w

[10] Das, R., Spence, G., Hogg, R. E., Stevenson, M. & Chakravarthy, U. Disorganization of inner retina and outer retinal morphology in diabetic macular edema. *JAMA Ophthalmol.* **136**, 202–208 (2018). 29327033 10.1001/jamaophthalmol.2017.6256 PMC5838716

[11] Kessler, L. J., Auffarth, G. U., Bagautdinov, D. & Khoramnia, R. Ellipsoid zone integrity and visual acuity changes during diabetic macular edema therapy: A longitudinal study. *J. Diabetes Res.* **2021**, 8117650 (2021). 34660813 10.1155/2021/8117650 PMC8516551

[12] Zhang, Y. P. et al. Artificial intelligence-driven radiomics study in cancer: The role of feature engineering and modeling. *Mil. Med. Res.* **10**, 22 (2023). 37189155 10.1186/s40779-023-00458-8 PMC10186733

[13] Liu, Z. et al. The applications of radiomics in precision diagnosis and treatment of oncology: opportunities and challenges. *Theranostics* **9**, 1303–1322 (2019). 30867832 10.7150/thno.30309 PMC6401507

[14] Lambin, P. et al. Radiomics: extracting more information from medical images using advanced feature analysis. *Eur. J. Cancer* **48**, 441–446 (2012). 22257792 10.1016/j.ejca.2011.11.036 PMC4533986

[15] Shahriari, M. H., Sabbaghi, H., Asadi, F., Hosseini, A. & Khorrami, Z. Artificial intelligence in screening, diagnosis, and classification of diabetic macular edema: A systematic review. *Surv. Ophthalmol.* **68**, 42–53 (2023). 35970233 10.1016/j.survophthal.2022.08.004

[16] Meng, Z. et al. Machine learning and optical coherence tomography-derived radiomics analysis to predict persistent diabetic macular edema in patients undergoing anti-VEGF intravitreal therapy. *J. Transl. Med.* **22**, 358 (2024). 38627718 10.1186/s12967-024-05141-7 PMC11022368

[17] Perez-Johnston, R. et al. CT-based radiogenomic analysis of clinical stage i lung adenocarcinoma with histopathologic features and oncologic outcomes. *Radiology* **303**, 664–672 (2022). 35230187 10.1148/radiol.211582 PMC9131171

[18] Lange, C., Feltgen, N., Junker, B., Schulze-Bonsel, K. & Bach, M. Resolving the clinical acuity categories “hand motion” and “counting fingers” using the Freiburg Visual Acuity Test (FrACT). *Graefes Arch. Clin. Exp. Ophthalmol.* **247**, 137–142 (2009). 18766368 10.1007/s00417-008-0926-0

[19] Zwanenburg, A. et al. The image biomarker standardization initiative: Standardized quantitative radiomics for high-throughput image-based phenotyping. *Radiology* **295**, 328–338 (2020). 32154773 10.1148/radiol.2020191145 PMC7193906

[20] Mayerhoefer, M. E. et al. Introduction to radiomics. *J. Nucl. Med.* **61**, 488–495 (2020). 32060219 10.2967/jnumed.118.222893 PMC9374044

[21] Wani, A. A. Comprehensive analysis of clustering algorithms: Exploring limitations and innovative solutions. *PeerJ Comput. Sci.* **10**, e2286 (2024). 39314716 10.7717/peerj-cs.2286 PMC11419652

[22] Efron, B. Bootstrap methods: Another look at the jackknife. *Ann. Stat.* **7**(1), 1–26 (1979).

[23] Lambin, P. et al. Radiomics: The bridge between medical imaging and personalized medicine. *Nat. Rev. Clin. Oncol.* **14**, 749–762 (2017). 28975929 10.1038/nrclinonc.2017.141

[24] Alloghani, M., Al-Jumeily, D., Mustafina, J., Hussain, A. & Aljaaf, A. J. A systematic review on supervised and unsupervised machine learning algorithms for data science 3–21 (2020).

[25] Seebock, P. et al. Unsupervised identification of disease marker candidates in retinal OCT imaging data. *IEEE Trans. Med. Imaging* **38**, 1037–1047 (2019). 30346281 10.1109/TMI.2018.2877080

[26] Sun, Q. et al. Biologic pathways underlying prognostic radiomics phenotypes from paired MRI and RNA sequencing in glioblastoma. *Radiology* **301**, 654–663 (2021). 34519578 10.1148/radiol.2021203281

[27] Yu, Y. et al. Magnetic resonance imaging radiomics predicts preoperative axillary lymph node metastasis to support surgical decisions and is associated with tumor microenvironment in invasive breast cancer: A machine learning, multicenter study. *EBioMedicine* **69**, 103460 (2021). 34233259 10.1016/j.ebiom.2021.103460 PMC8261009

[28] Deák, G. G. et al. A systematic correlation between morphology and functional alterations in diabetic macular edema. *Investig. Ophthalmol. Vis. Sci.* **51**, 6710–6714 (2010). 20484587 10.1167/iovs.09-5064

[29] Sil Kar, S. et al. Multi-compartment spatially-derived radiomics from optical coherence tomography predict anti-VEGF treatment durability in macular edema secondary to retinal vascular disease: Preliminary findings. *IEEE J. Transl. Eng. Health Med.* **9**, 1000113 (2021). 34350068 10.1109/JTEHM.2021.3096378 PMC8328398

[30] Baharlouei, Z., Rabbani, H. & Plonka, G. Wavelet scattering transform application in classification of retinal abnormalities using OCT images. *Sci. Rep.* **13**, 19013 (2023). 37923770 10.1038/s41598-023-46200-1 PMC10624695

[31] Wu, Q. et al. Detection of morphologic patterns of diabetic macular edema using a deep learning approach based on optical coherence tomography images. *Retina* **41**(5), 1110–1117 (2021). 33031250 10.1097/IAE.0000000000002992 PMC8078116

[32] De Fauw, J. et al. Clinically applicable deep learning for diagnosis and referral in retinal disease. *Nat. Med.* **24**(9), 1342–2135 (2018). 30104768 10.1038/s41591-018-0107-6

